# Synthesis and Characterization of Poly(1,4,7-trioxacycloundecane-8,11-dione) Macrocyclic Functionalized Hydrogel for High Selectivity Adsorption and Complexation of Bismuth Ion

**DOI:** 10.3390/polym10060662

**Published:** 2018-06-13

**Authors:** Brian A. Omondi, Hirotaka Okabe, Yoshiki Hidaka, Kazuhiro Hara

**Affiliations:** 1 Department of Applied Quantum Physics and Nuclear Engineering, Faculty of Engineering, West 2, 922-2, Kyushu University, 744 Motooka, Fukuoka 819-0395, Japan; okabe@ap.kyushu-u.ac.jp (H.O.); hidaka@ap.kyushu-u.ac.jp (Y.H.); hara.kazuhiro.590@m.kyushu-u.ac.jp (K.H.); 2 Center for Research and Education of Environmental Technology, Faculty of Engineering, Kyushu University, 744 Motooka, Fukuoka 819-0395, Japan

**Keywords:** bismuth, adsorption, macrocyclic hydrogels, host-guest complexation, sol-gel synthesis, molecular recognition technique

## Abstract

Macrocyclic functional hydrogels incorporating new poly cyclic active sites (1,4,7-trioxacycloundecane-8,11-dione) within their entire network, have been synthesized. Using the high-dilution coupling of the bi-functional monomers maleic acid and bis(chloroethyl)ether in a sol-gel chemistry synthesis, 11-membered chelate rings infused with three oxygen donor atoms were created and characterized, and their structures confirmed using Fourier transform infrared (FTIR) and nuclear magnetic resonance (NMR) spectroscopic analyses. The macrocyclic gel, designed for selective host-guest adsorption and complexation of metal substrates, was initially tested against an aqueous set of 14 metal competitive solutions, where it demonstrated exclusive selectivity for Bi^3+^_aq_, with the other metals exhibiting zero adsorption. Further analysis using binary and single ion Bi^3+^-containing solutions showed a near-complete removal of Bi^3+^ using this polycyclic hydrogel, with 98% extraction efficiency and *q* = 9.80 mg/g. These results clearly confirm that the 1,4,7-trioxacycloundecane-8,11-dione cyclic sites are most suitable for high selectivity and capture of Bi. The metal substrates were entrapped within the 1,4,7-trioxacycloundecane-8,11-dione cyclic sites. Evidently, by exploiting the host-guest complexation chemistry of macrocycles, we were able to design hydrogel adsorbents whose networks were comprised entirely of macrocyclic active groups for possible purification works of copper involving bismuth impurities, and/or for efficient selective uptake and recovery of bismuth trace ions existing in highly competitive environments such as sea water.

## 1. Introduction

While there have been various methods for the capture and removal of heavy metals, adsorption has remained the method of choice in research efforts. This owes to the simplicity, versatility, and effectiveness that can be derived from the various adsorbents, such as zeolites [1,2,3,4], activated carbons, biomaterials [5], carbon nanotubes [6], and recently, hydrogels [7,8,9]. Adsorbents also afford the much-desired ability to recover the metals and other adsorbates for potential reuse [10]. This is a standout property that greatly subsidizes the cost, since in typical areas such as heavy metal decontamination of waste waters, if effectively isolated within the adsorbent and later recovered, the adsorbed metals can still be useful for secondary re-application. This makes the whole adsorption process cost-effective.

However, most adsorbents rely on mechanisms which are not very restrictive in their selectivity during the adsorption process. For example, the adsorption of heavy metals via ion exchange [1,3,5,11] is a general phenomenon which proceeds via the substitution of target substrates with exchangeable ions of similar charge on the adsorbent surface. Generally speaking, all metals are positively charged and would therefore participate in ion exchange and would only vary based on charge density. This renders the ion exchange mechanism unreliable where selectivity is paramount for distinguishing substrates in a competing environment. It also creates a gaping need that requires extending of the adsorbents’ effectiveness by incorporating new functional groups that can instead exploit more inherently specific and characteristic properties of substrates, to ensure that it is only the target substrates that are then captured, and this way we achieve adsorption selectivity. 

Emerging adsorbent materials therefore have tended to use macrocyclic groups, which are then grafted onto solid adsorbent supports in order to facilitate selective adsorption. This is because macrocyclic groups come as enclosed zones which are then used to entrap substrates such as metal ions in a host-guest mechanism [12] based on size and shape. Host-guest adsorption exploits characteristic morphological properties of substrates to separate and adsorb only the substrates which can fit into the macrocyclic cavities. This phenomenon was adopted from biological macrocyclic chelators [13,14] and supramolecular chemistry [15,16,17]. Attempts to translate this mechanism onto adsorbents have not been very successful, with few exceptions (e.g., the SuperLig^®^ adsorbent) [18,19,20]. SuperLig^®^ works as an ion exchange resin which utilizes a poly(hydroxyarylene) ligand (grafted onto zeolite base support) as a form of molecular recognition technique for high-affinity cesium adsorption. However, SuperLig^®^ only partially solves the problem, since it remains limited as an ion exchanger, and not a fully macrocyclic material exploiting a host-guest complexation mechanism. Secondly, grafting creates a dependency problem in that the macrocyclic groups to be grafted are limited by the type of solid support which can enable grafting, the relevant grafting technique to employ, and the fact that macrocyclic groups to be grafted must be prepared separately and in a form that can lead subsequent grafting onto the solid supports. This increases uncertainty in an already hurdled path, which has subsequently curtailed the development of macrocyclic adsorbents. Grafting also prevents full exploitation of the active groups, since the majority of the adsorbent will be made up of the solid support holding the grafted groups, instead of the latter. 

In view of these facts, in our initial attempts to develop a polycyclic functional hydrogel [21], we successfully described a method in which adsorbents in the form of a hydrogel can be prepared as a fully macro-cyclized material, eliminating any need for grafting. Through sol-gel synthesis, eight-membered shaped polycyclic groups infused with nitrogen donor atoms were fabricated. These groups transcended the entire hydrogel network, creating a three-dimensional material which revealed selectivity for Fe^3+^_aq_ and metal ions with sizes between 1.23–1.29 Å. This direct synthesis of macrocyclic hydrogel adsorbent opened up possibilities for altering the ring nature (size, shape, type, and number of donor atoms) for creating more adsorbents with different selectivity and stronger adsorption capacity for target cations. 

The present work explored the fabrication of a new type of macrocyclic hydrogel adsorbent. We report a synthesis method via sol-gel polymerization and cross-linking that utilizes new bi-functional monomers, maleic acid and bis(chloroethyl)ether, to create a larger cyclic ring with more donor atoms. Bis(chloroethyl)ether monomer was selected to contribute three oxygen (O) donor atoms into the macrocyclic ring and thereby increase the chelation power and hence the degree of adsorption of the functional hydrogel. Thus, by altering the ring size, its shape, the type and number of donor atoms, and thereafter replicating the newly formed rings via polymerization and cross-linking into a 3D hydrogel network, we anticipated the synthesis of a new type of macrocyclic hydrogel adsorbent with possibly different selectivity than previous work. Therefore, herein we report the synthesis, characterization, and adsorption performance of this new type of adsorbent. 

## 2. Materials and Methods

### 2.1. Materials

Maleic acid and bis(chloroethyl)ether ligand monomers were obtained from Sigma Aldrich (Ishikari Hokkaido, Japan), including polymerization agents ammonium persulfate (APS) and *N*,*N*′-methylenebisacrylamide (NMBA). Acrylic acid was sourced from Wako chemicals (Osaka, Japan). The main solvent in all references was ultrapure deionized water. Metal ion stock solutions (Bi^3+^, Cu^2+^, and 14-metal multi-element solution) were all purchased from Merck (Darmstadt, Germany). All chemicals were of high analytical grade, and as such were used as received without further purification. 

### 2.2. Hydrogel Adsorbent Fabrication via Sol-Gel Method

The first stage in adsorbent preparation involved coupling equimolar amounts (1 M each) of maleic acid and bis(chloroethyl)ether monomers under high aqueous dilution following the method of Stetter et al. [22]. The mixture was then immediately heated at sustained temperatures of 90 °C for 10 min in order to obtain a cyclic precondensate product, which formed the pregel solution. A small amount of acrylic acid (0.2 M) was added to this pregel product in order to promote polymerization into polycyclic chains, through the acrylate vinyl radical groups which have a high propensity for instigating polymerization. Finally, 0.05 M NMBA cross-linking agent and 0.005 M APS polymerization initiator were added respectively in order to instigate the sol-gelation process. The final solution was incubated at 50 °C isothermal temperature in a water bath for 20 h, and maintained at these polymerization conditions, following which we obtained a hydrogel product, hereon labeled “BCE hydrogel”. 

### 2.3. Hydrogel Processing

**Figure 1:**
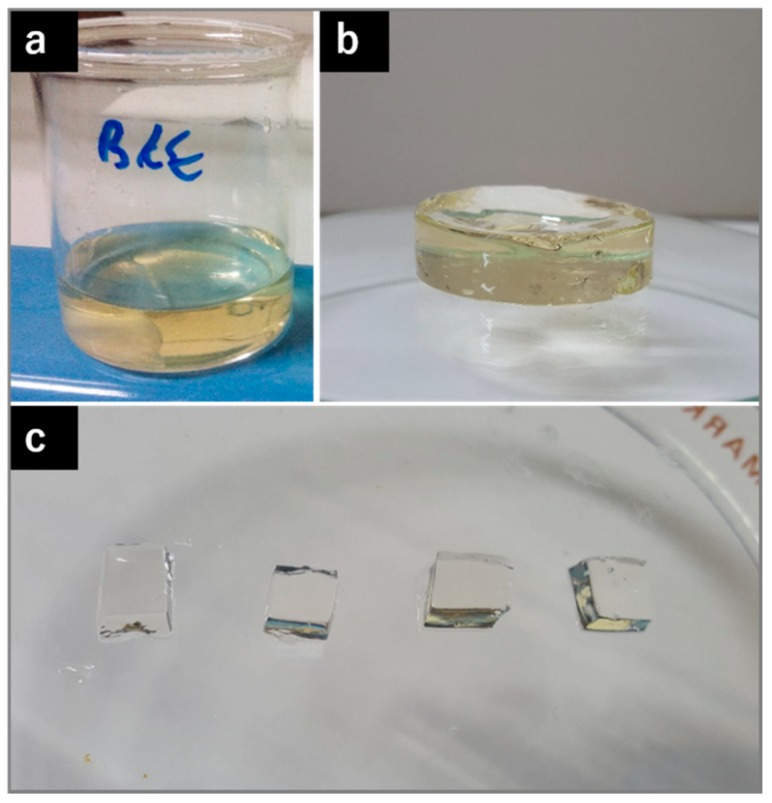
(**a**) Newly fabricated BCE hydrogel; (**b**) extracted hydrogel; and (**c**) hydrogel fractions.

Newly fabricated BCE hydrogel was extracted from the synthesis bottles and processed into small predetermined cubed fractions (0.5 × 0.5 × 0.5 cm^3^) as summarized in Figure 1. These pieces were then washed in water for a prolonged period (40 h) in order to elute all unreacted monomers and also dissolve any oligomers formed during the synthesis process. The final washed product was therefore the cross-linked and undissolved adsorbent material, which was then dried until constant weight. Hydrogel equilibrium swelling in distilled deionized water revealed a swelling capacity of X73 of its initial dry weight, whereas the gel fraction was determined as 64% using Equation (1):

(1) $$\mathrm{Gel}\;\mathrm{fraction}\;G.F=(\frac{W_{o}-W_{d}}{W_{o}})\times 100,$$

where $W_{o}$ is the dry weight (g) of unwashed newly synthesized material; $W_{d}$ is the dry weight of washed hydrogel. Dried samples were later characterized using Fourier transform infrared (FTIR) and nuclear magnetic resonance (NMR) spectroscopic techniques to determine BCE hydrogel’s surface features, functional groups identification, among other properties prior to final application against metal ion adsorption. 

### 2.4. Adsorption Studies 

#### 2.4.1. Multi-Element Adsorption 

**Scheme 1:**
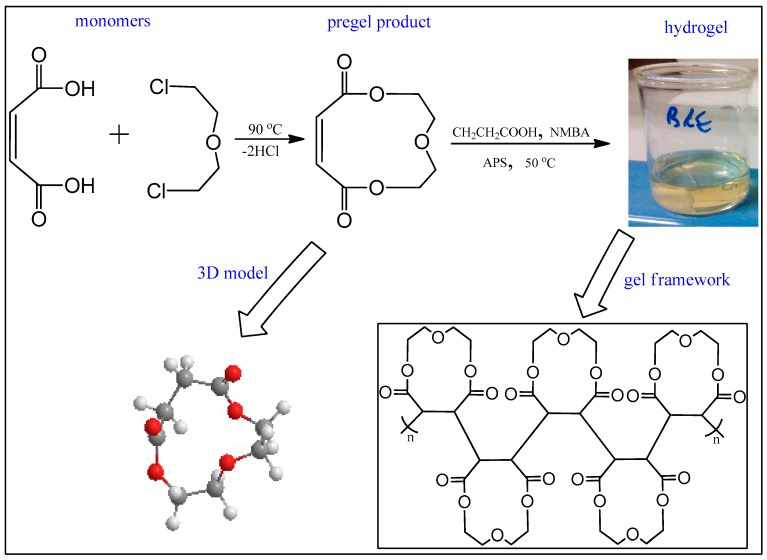
Proposed synthesis mechanism for BCE hydrogel adsorbent.

Using dried BCE hydrogel samples as the main adsorbent, the polycyclic 1,4,7-trioxacycloundecane-8,11-dione functional units were tested for adsorption performance and selectivity of aqueous metal ions solutions. The dione active site was an 11-membered ring with three oxygen donor atoms. Its ring shape and size/morphology as typified in the 3D model in Scheme 1 can only accommodate one or few types of cations during complexation. Therefore, we screened for the type of cation selective for this active site. 

For this process, we used an integrated aqueous 14-metal multi-element solution of 0.01 L each. The multi-element solution was made up of aqueous metal ions of +1, +2, and +3 ionic charges. Some were group 1 metals, others transition metals, while there were also post-transition metal ions. 

The initial concentrations of this solution were fixed at 250, 500, and 1000 ppb for each of the respective constituent metal ions making up the multi-element solution to also find out the effect of change in concentration. Into these three solutions, hydrogel adsorbents of 0.02 g were immersed for batch adsorption experiments for up to 20 h, under constant agitation of 130 rpm at ambient temperatures. After adsorption, the gel adsorbents were extracted from these solutions and the residue solution measured for the change in concentration using an Agilent ICP/MS instrument (Tokyo, Japan).

#### 2.4.2. Bi and Bi/Cu Adsorption Studies

To fully test the effectiveness of 1,4,7-trioxacycloundecane-8,11-dione macrocyclic sites as ideal for selective Bi^3+^ adsorption, we aimed to optimize various adsorption conditions. Accordingly, single solutions of aqueous Bi^3+^ and binary Bi/Cu solutions were used for this investigation. Keeping the test solution volumes constant at 0.01 L in all cases, we increased the starting concentrations of Bi^3+^ solution progressively between 10–60 mg/L in batch adsorption experiments. For each solution, the total amount of BCE hydrogel used was varied, and the adsorption time was also changed gradually to study adsorption kinetics. At the same time, binary solutions of Bi/Cu were used in alternating concentrations to study competitive adsorption between these two ions. 

After the adsorption process, the residual concentration of the adsorbed solution in all cases was analyzed using an ICP/MS instrument and the obtained values were used to determine parameters such as the adsorption capacity (*q*) and percent removal (%_AD_) based on Equations (2) and (3), respectively:

(2) $$q=\left(\frac{C_{0}-C_{e}}{m}\right)\times V,$$

(3) $${\%}_{AD}=\left(\frac{C_{0}-C_{e}}{C_{0}}\right)\times 100,$$

where *q* (mg/g) is the adsorption capacity, *C*_0_ and *C*_e_ are the starting concentration and the post-adsorption equilibrium concentrations (mg/L), respectively; *m* is the adsorbent mass (g) and *V* is the analyte volume (L).

## 3. Results and Discussion

### 3.1. Characterization of BCE Hydrogel

Conventional spectroscopic techniques employed to establish, analyze, and confirm this functional group included FTIR and ^13^C NMR methods.

#### 3.1.1. FTIR Analysis

FTIR analyses of solid dried samples were undertaken via the Jasco 4100 instrument (Tokyo, Japan) using the attenuated total reflectance (ATR) mode. Absorption and transmittance measurements were carried out for each freshly synthesized sample, and the obtained wavenumbers were analyzed to reveal possible functional groups within the BCE hydrogel. 

**Figure 2:**
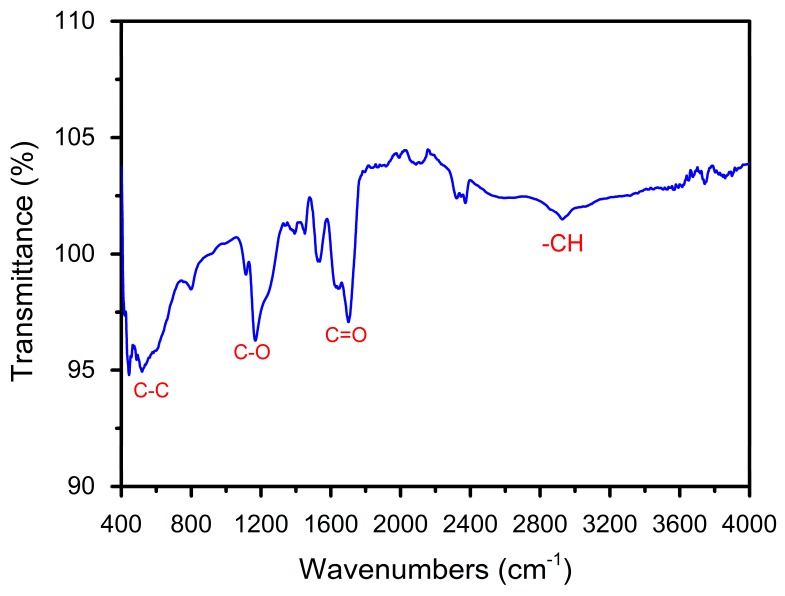
Fourier transform infrared (FTIR) spectral analysis of BCE hydrogel adsorbent.

The results in Figure 2 revealed that four main vibrational peaks constituted the active sites of the BCE hydrogel. 

From Figure 2, we could see that various combinations C, H, and O appendages formed the main functional groups that transversed the entire network of the BCE hydrogel active sites. C–H and C=O stretching vibrations were isolated at 2900 and 1700 cm^−1^, respectively. A corresponding stretch for the C–O group appeared at 1090 cm^−1^, whereas the bending vibration of C–C group was characteristic in the fingerprint region of 550 cm^−1^. Identification of these groups necessitated a further study using the JOEL JNM-ECX 400 ^13^C NMR spectroscopy (Tokyo, Japan) in order to establish how these groups were arranged and connected into a full and coherent chemical structure. 

#### 3.1.2. ^13^C NMR

**Figure 3:**
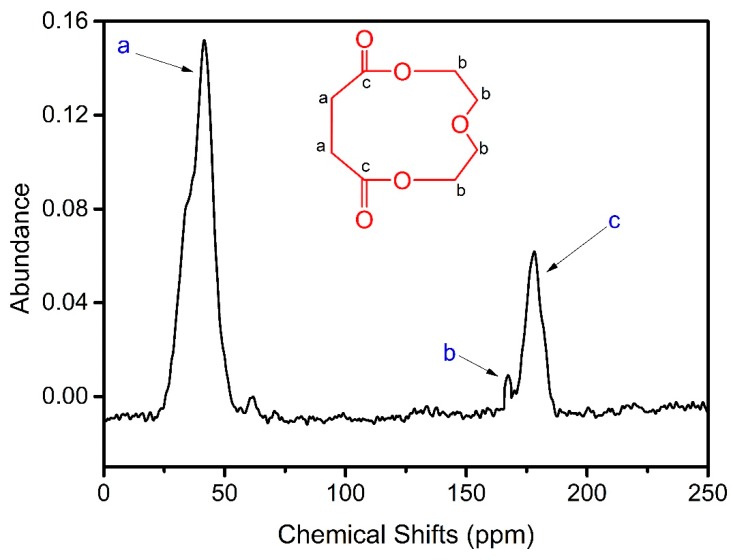
^13^C nuclear magnetic resonance (NMR) spectral analysis of BCE hydrogel adsorbent.

^13^C NMR analysis was undertaken to establish the types of equivalent carbons in the BCE hydrogel using the cross-polymerization magic angle spinning (CPMAS) technique. This is a high-resolution solid state technique for improving the S/N ratio and minimizing large anisotropic NMR interactions in order to ensure better spectra. According to the results in Figure 3, only three equivalent carbons could be identified from the NMR spectroscopy. Existence of only three equivalent carbons alluded that BCE hydrogel, despite being a complex cross-linked polymeric material, could contain only a single type of functional group, which is replicated and connected in the same way throughout the structure so as to appear simplistic enough to yield only three simple carbon NMR peaks. 

Evidently, based on the ^13^C NMR results, the main groups previously isolated in the FTIR results should then form part of these three types of carbon. 

As shown in the inset of Figure 3, the proposed functional unit of BCE hydrogel is a 1,4,7-trioxacycloundecane-8,11-dione monomer unit. This structural unit was comprehensively summarized by the NMR chemical shift results. In the ^13^C NMR findings, carbon type ***a*** was the least-deshielded carbon, since it is connected to sp^2^ carbon at either end. Therefore, its chemical shifts would appear downfield at 42 ppm. However, carbon type ***b*** is found in a different chemical environment: for each of the four carbons, on one end it is connected to an sp^2^ carbon, but at the opposite end it is heavily deshielded by an oxygen atom. Since this is common to all four, they all resonate as one equivalent carbon which appeared upfield at 167 ppm due to the electron withdrawal effect of oxygen. Finally, carbon type ***c*** is a carbonyl carbon, which experiences the deshielding effect of a double bond group in addition to a carbonyl oxygen atom; their overall contribution caused a most upfield resonation at 178 ppm. These ^13^C NMR analytical results were akin to similar discussion by Omondi et al. [21] regarding the polycyclic (1,4-diazocane-5,8-dione) hydrogel. 

In the present discussion, the obtained ^13^C NMR results also perfectly correlated with earlier FTIR findings, where the C=O, C–O, C–C, and C–H (ref: carbons ***a*** and ***b***) functional groups were clearly depicted as inherent of the cyclic ring. Ultimately, this implied that 1,4,7-trioxacycloundecane-8,11-dione was the main monomer unit, which was polymerized and cross-linked to form the BCE hydrogel adsorbent. 

#### 3.1.3. BCE Hydrogel Chemistry

These spectroscopic results ultimately confirmed our earlier premise that double condensation of maleic acid and bis(chloroethyl)ether monomers (under high temperatures and dilution conditions) formed a precondensate cyclic ring with the elimination of HCl molecules as byproducts during the high-temperature condensation coupling. The precondensate, through its double bond sites, underwent polymerization and cross-linking into a three-dimensional BCE hydrogel material as summarized in Scheme 1. Previous works with maleic acid [21] and the successfully synthesized hydrogel herein corroborate this synthesis route for macrocyclic hydrogels. 

Looking at Scheme 1, we posited that BCE hydrogel comprises an 11-membered cyclic group, which is repeatedly polymerized and cross-linked into a solid macrocyclic material using NMBA cross-linking agent. 

### 3.2. Adsorption Studies

#### 3.2.1. Multi-Element Adsorption 

**Figure 4:**
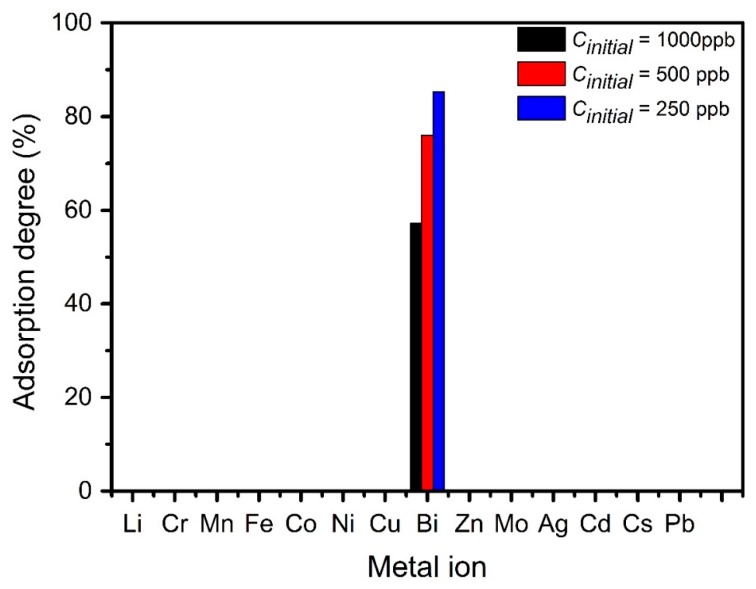
Adsorption from multi-element competitive aqueous solution showing exclusive adsorption of Bi^3+^ with zero uptake of other competing cations.

Preliminary results illustrated in Figure 4 indicated that in all cases, BCE hydrogel only adsorbed the bismuth (Bi^3+^) ion from the entire 14-metal ion competitive solution. The other metals achieved zero adsorption, with no change in initial concentration. In other words, despite the high level of metal-metal competition, the BCE hydrogel demonstrated an acutely high propensity for exclusive Bi^3+^ adsorption at all levels of concentration and adsorption conditions. 

In Figure 4, Bi^3+^ adsorption capacity increased between 60–87% depending on the level of initial concentrations. For lower-concentrated solutions, the adsorbent was able to adsorb nearly all the metal ions due to the large number of available active sites to accommodate entrap and complex the Bi^3+^ ions. However, when the initial solution concentration was increased, this also increased the number of ions competing for fewer available adsorption sites, leading to a lower percent extraction (67%) once all the sites were occupied. Additionally, the other 13 non-adsorbed ions existing within this solution could cause a crowding effect which reduces accessibility of Bi^3+^ onto the active sites, thus contributing to lower adsorption capacity. 

In short, the high adsorption selectivity achieved at this stage demonstrated that the 1,4,7-trioxacycloundecane-8,11-dione macrocyclic sites are especially fashioned for Bi^3+^ capture. Adsorption likely occurred within these cyclic sites in a host-guest lock mechanism, where only Bi^3+^ substrates were found to most favorably complement the dione macrocyclic sites, and thus could be aptly accommodated therein. 

**Scheme 2:**
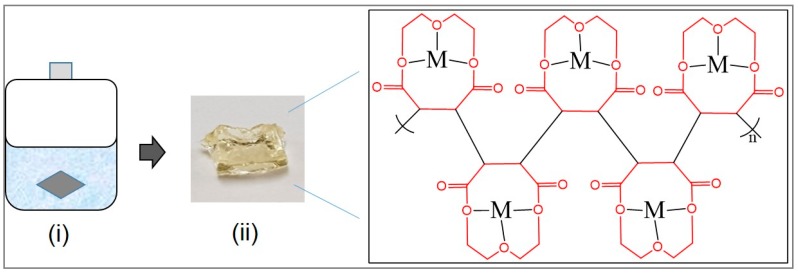
Bismuth complexation by BCE hydrogel’s macrocyclic active sites {M = [Bi(H_2_O)_8_]^3+^}.

Bi^3+^_aq_ is a trivalent metal ion with a solvation number of 8 in aqueous solution, existing in a square antiprism configuration, as has been extensively studied by Persson et al. [23,24]. Further, Persson also found out that this aqua ion has an ionic radius of 1.07 Å. Therefore, in the context of our results so far, we could posit that the tendency of BCE hydrogel macrocyclic sites for exclusive bismuth adsorption points towards these cyclic sites’ high preference for substrates of octahedral aqua ions with square antiprism configuration, and having a radial diameter size of 2.14 Å for host-guest complexation (demonstrated in Scheme 2). Earlier, Cram et al. [25] when discussing macrocyclic polyethers, was also able to demonstrate the cation binding and ring encapsulation property of macrocyclic rings which acts in a similar way as proposed for the BCE hydrogel herein. 

Following such impressive results of Bi^3+^ selectivity from multi-ion competitive solution, this phenomenon was further analyzed using single and binary solutions of Bi^3+^. 

#### 3.2.2. Bi and Bi/Cu Adsorption Studies

Bismuth is one of the undesirable yet difficult-to-remove impurities during copper mining processes [26,27,28]. Its effective removal demands techniques that should only isolate and remove bismuth without altering copper quantities. In this investigation, we initially studied the optimum properties of BCE gel for single ion solutions of Bi^3+^, and thereafter proceeded to apply these parameters against binary solutions of Bi/Cu to simulate typical conditions involving bismuth as a copper impurity. 

##### Results on Adsorbent Mass-Analyte Concentration Correlation 

**Figure 5:**
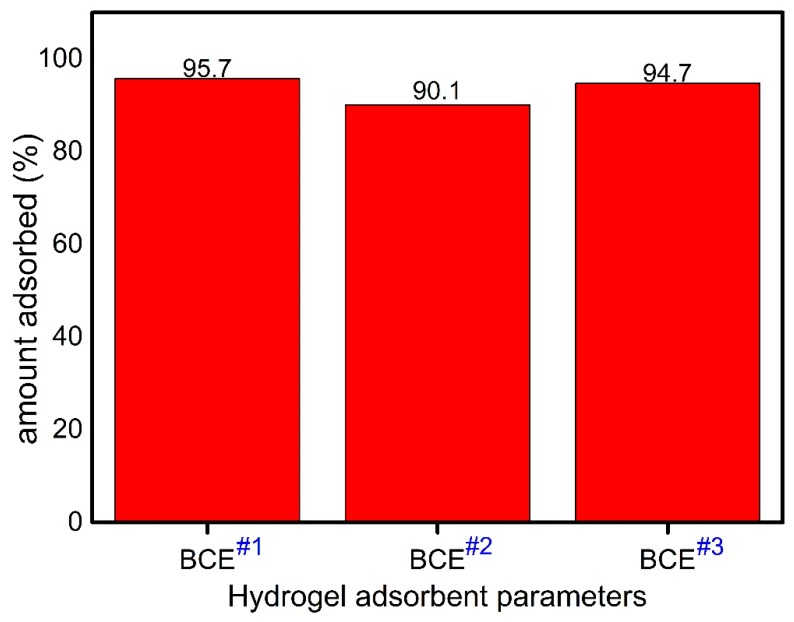
Bismuth adsorption from non-competitive aqueous solution.

Against single ion solutions, by varying the initial concentration and the amount of adsorbents for each type of solution, the authors found that in order to achieve maximum Bi^3+^ adsorption, BCE hydrogel mass needed to be chosen critically relative to the concentration and volume of the analyte. This is outlined in Table 1, and the results are summarized in Figure 5. 

From the results in Figure 5, the degree of initial concentration of the metal solution directly correlated with the amount of BCE hydrogel adsorbent to be applied in order to achieve optimum equilibrium adsorption capacities of at least 95% of total concentrate. Gel amount and initial concentration obeyed an ideal ratio of 1:1 to give the best degree of adsorption, as seen in columns BCE#1 and BCE#3 in Figure 5. The relation is summarized as follows:

$$\frac{concentration}{gel\;amount}=\frac{20\;\mathrm{mg}/L}{20\;\mathrm{mg}}=\frac{40\;\mathrm{mg}/L}{40\;\mathrm{mg}}=\frac{1}{1}$$

However, if a different ratio was used (e.g., for BCE#2 where we had 1:2, which was a slight deviation from the optimal 1:1 ratio), this consequently led to a lower adsorption capacity with only 90% efficiency attained, instead of the expected 95%. 

Guided by these results, we proceeded to analyze the degree of Bi^3+^ removal at various starting concentrations, all the while maintaining the ideal ratio of 1:1. These studies were extended to Bi/Cu competitive adsorption due to these metals’ co-existence, so as to establish the influence of their interaction on the nature of BCE hydrogel adsorption. The two metals were used at alternating high concentrations within the same solution, while in the third solution they were put at equal starting concentrations, in order to simulate three different scenarios. This is elaborated in Table 2. However, since these two metals co-existed in the same solution at different concentrations, for binary adsorption it was not possible to ensure gel mass-to-concentration ratio at 1:1. Instead, we used an average gel mass for each case, which would be closer to this ratio. 

##### Gel Performance against Single and Binary Solutions 

**Figure 6:**
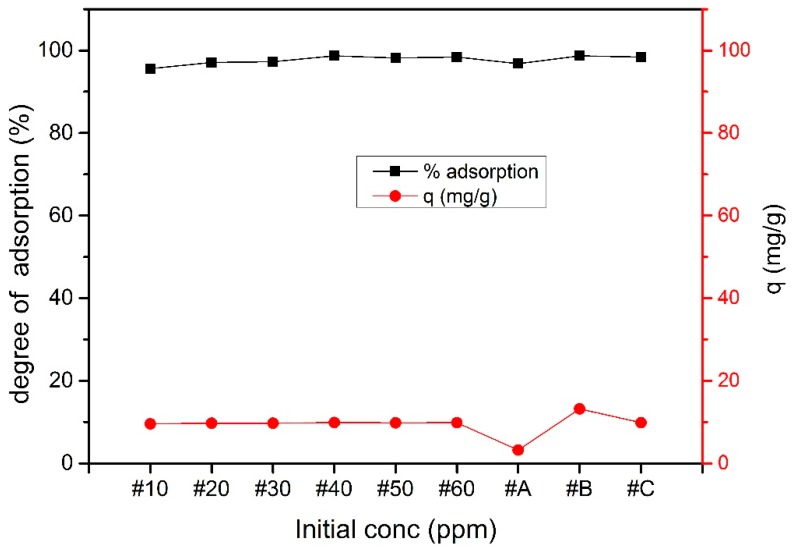
Adsorption performance of BCE hydrogel against single-ion aqueous Bi solutions and binary solutions of: #A = Bi10/Cu40 mg/L; #B = Bi40/Cu10 mg/L; and #C = Bi40/Cu40 mg/L.

In the adsorption performance results highlighted in Figure 6, with original concentrations of Bi^3+^ ranging between 10–60 mg/L, and the gel-to-analyte ratio maintained at 1:1, we found that almost all of the Bi^3+^ ions were completely adsorbed onto the hydrogel active sites, with an average 98% removal efficiency from the aqueous solutions. Further, there was zero adsorption of Cu in all cases of binary solutions, with Cu concentration remaining unchanged even at prolonged adsorption periods. This further demonstrated BCE hydrogel’s fingerprint adsorption and proclivity towards only Bi^3+^. 

Moreover, we could gather from Figure 6 that the actual gel adsorption capacities were remarkably high, showing approximate values of 9.80 mg/g from analyte volumes of 10 mL. However, in binary solutions of Bi^3+^ and Cu where we used an average hydrogel mass of 0.03 g (which deviated from the ideal ratio of 1:1), Figure 6 shows that for these two cases, the adsorption capacities consequently deviated, giving slightly lower values. 

Nevertheless, this high capture and selective adsorption for Bi^3+^ emphasized the effectiveness of cyclic active sites in metal capture adsorbents, and the mechanism as akin to molecular recognition technique. The BCE hydrogel polycyclic sites’ ring size and shape are fashioned to complement the morphological dimension of only the target substrate (bismuth), leading to profound exclusive capture of only those specific substrates, with high adsorption capacities. Additionally, adsorption was enhanced via chelation of bismuth analyte by the macrocyclic donor atoms, which ensured that the captured substrates were immobilized within the ringed sites, to prevent their loss or prevent them from displacement by other substrates in the case of competition. 

Recalling earlier results against the 14-metal multi-element solution, the degree of Bi^3+^ adsorption and removal ranged between 60–87%. However, here in Figure 6 when the competition was reduced to contain a binary pair of Bi/Cu, or if competition was entirely eliminated as in the case of single solutions, we achieved adsorption efficiencies of at least 98%. It was therefore likely that for the high competitive 14-metal multi-element solution, the other constituent metals caused an overbearing effect on Bi^3+^, preventing ease of accessibility of the latter into the 1,4,7-trioxacycloundecane-8,11-dione macrocyclic sites. This crowding effect possibly explained the slightly reduced adsorption capacity in the 14-metal competitive solution compared to binary and single solutions where there was little or no crowding. But in all cases, the integrity of Bi^3+^ specificity by the gel active sites was maintained, with other metals experiencing zero adsorption onto the 1,4,7-trioxacycloundecane-8,11-dione active sites. 

## 4. Conclusions

BCE hydrogel was successfully synthesized and characterized using spectroscopic methods to reveal a 1,4,7-trioxacycloundecane-8,11-dione macrocyclic group as the main active site. The hydrogel’s uniqueness stems from the fact that macrocyclic sites were directly synthesized using a double condensation method, and then propagated to populate the entire structure of a hydrogel using a sol-gel method without any grafting, such that the entire hydrogel contained cross-linked networks of 1,4,7-trioxacycloundecane-8,11-dione macrocyclic groups. Since the design of this group revealed a special proclivity and exclusivity towards bismuth aqua ion, the resultant hydrogel was therefore a more potent adsorbent for bismuth cation. Against single, binary, and multi-element competitive solutions, BCE hydrogel was uniquely selective against Bi^3+^ adsorption, irrespective of metal-metal competitions, with 98% extraction efficiencies. These results ultimately highlighted the effectiveness of the macrocyclic hydrogel which uses a form of molecular recognition technology for selective adsorption by exploiting the inherent and distinguishing characteristics of target substrates to achieve high adsorption specificity. This phenomenon could also be important for applications which require effective extraction of bismuth which exists as impurities during copper mining processes. This hydrogel could also be applied as technology for sea water extraction and harvesting of bismuth by overcoming the high degree of competition found in sea water. Other potential applications include catalysis, which requires fingerprint impregnation of specific metals onto a material structure. By designing a catalyst framework to include macrocyclic sites such as 1,4,7-trioxacycloundecane-8,11-dione, target metals such as Bi or others could be effectively deposited onto these sites embedded within solid structural supports. The authors declare no conflict of interest in the preparation of this research work. 

## Figures and Tables

**Table 1:** Adsorption parameters for non-competitive adsorption of Bi^3+^.

Parameters	#1	#2	#3
BCE Hydrogel amount	0.02 g	0.04 g	0.04 g
Bi_(aq)_ initial conc.	20 mg/L	20 mg/L	40 mg/L
Solution volume	10 mL	10 mL	10 mL

**Table 2:** Parameters for BCE hydrogel amounts and analyte concentrations for adsorption from binary metal ion competitive solution.

Bi/Cu Aqueous binary solution		BCE Gel amount (g)
Bi (mg/L)	Cu (mg/L)	
10	40	0.03
40	10	0.03
40	10	0.04
